# Supplementary material for: Interhemispheric Functional Connectivity in the Primary Motor Cortex Assessed by Resting-State Functional Magnetic Resonance Imaging Aids Long-Term Recovery Prediction among Subacute Stroke Patients with Severe Hand Weakness
Source: J Clin Med. 2020 Apr 1;9(4):975. doi: 10.3390/jcm9040975 (PMC7230262; doi:10.3390/jcm9040975)
Supplement: Supplementary file 1 [file jcm-09-00975-s001.pdf]

Table S1. Individual ROI coordinates obtained from task-fMRI.

Subject	Group	Right M1 MNI coordinates			Left M1 MNI coordinates			Right SMA MNI coordinates			Left SMA MNI coordinates		
		x	y	z	x	y	z	x	y	z	x	y	Z
1	Good	46	-16	58	-33	-24	48	8	-8	56	-8	-8	56
2	Good	44	-22	58	-34	-24	60	8	-8	56	-8	-8	56
3	Good	52	-17	52	-48	-23	54	8	-8	56	-8	-8	56
4	Good	59	-37	42	-48	-25	53	8	-8	56	-8	-8	56
5	Good	34	-22	46	-42	-25	58	8	-8	56	-8	-8	56
6	Good	38	-30	56	-34	-24	54	8	-8	56	-14	-9	62
7	Good	38	-20	56	-34	-24	58	8	-8	56	-8	-8	56
8	Good	34	-19	56	-35	-20	60	8	-8	56	-8	-8	56
9	Good	36	-22	52	-36	-22	52	10	4	52	-8	-8	52
10	Good	36	-14	56	-28	-22	54	6	0	52	-10	-4	52
11	Good	38	-28	70	-52	-18	48	10	-8	68	-10	-2	64
12	Poor	34	-28	52	-44	-36	58	8	-8	56	-8	-8	56
13	Poor	53	-36	58	-38	-38	55	8	-8	56	-8	-8	56
14	Poor	31	-32	46	-33	-28	54	10	6	58	-8	-8	56
15	Poor	40	-26	46	-44	-28	60	8	-8	56	-8	-8	56
16	Poor	38	-26	42	-34	-24	60	9	4	52	8	-8	56
17	Poor	33	-22	54	-34	-24	60	8	-8	56	-8	-8	56
18	Poor	36	-23	62	-40	-26	60	8	-8	56	-8	-8	56
19	Poor	48	-30	58	-32	-28	61	8	2	56	-8	-8	56
20	Poor	42	-14	58	-34	-28	56	8	-8	56	-8	-14	62
21	Poor	40	-24	58	-32	-30	60	10	-14	64	-10	-14	64
22	Poor	42	-22	66	-38	-26	60	8	-12	60	-4	6	66
